# Quantitative Real-Time Analysis of Differentially Expressed Genes in Peripheral Blood Samples of Hypertension Patients

**DOI:** 10.3390/genes13020187

**Published:** 2022-01-21

**Authors:** Fawad Ali, Arifullah Khan, Syed Aun Muhammad, Syed Shams ul Hassan

**Affiliations:** 1 Riphah Institute of Pharmaceutical Sciences, Riphah International University, Islamabad 44000, Pakistan; fawad.alee@gmail.com (F.A.); arif.ullah@riphah.edu.pk (A.K.); 2 Department of Pharmacy, Kohat University of Science and Technology, Kohat 26000, Pakistan; 3 Institute of Molecular Biology and Biotechnology, Bahauddin Zakariya University, Multan 60800, Pakistan; 4 Shanghai Key Laboratory for Molecular Engineering of Chiral Drugs, School of Pharmacy, Shanghai Jiao Tong University, Shanghai 200240, China; 5 Department of Natural Product Chemistry, School of Pharmacy, Shanghai Jiao Tong University, Shanghai 200240, China

**Keywords:** cDNA datasets, hypertension, differentially expressed genes, enrichment analysis, qPCR, expression profiling

## Abstract

Hypertension (HTN) is considered one of the most important and well-established reasons for cardiovascular abnormalities, strokes, and premature mortality globally. This study was designed to explore possible differentially expressed genes (DEGs) that contribute to the pathophysiology of hypertension. To identify the DEGs of HTN, we investigated 22 publicly available cDNA Affymetrix datasets using an integrated system-level framework. Gene Ontology (GO), pathway enrichment, and transcriptional factors were analyzed to reveal biological information. From 50 DEGs, we ranked 7 hypertension-related genes (*p*-value < 0.05): ADM, ANGPTL4, USP8, EDN, NFIL3, MSR1, and CEBPD. The enriched terms revealed significant functional roles of HIF-1-α transcription; endothelin; GPCR-binding ligand; and signaling pathways of EGF, PIk3, and ARF6. SP1 (66.7%), KLF7 (33.3%), and STAT1 (16.7%) are transcriptional factors associated with the regulatory mechanism. The expression profiles of these DEGs as verified by qPCR showed 3-times higher fold changes (2−ΔΔCt) in ADM, ANGPTL4, USP8, and EDN1 genes compared to control, while CEBPD, MSR1 and NFIL3 were downregulated. The aberrant expression of these genes is associated with the pathophysiological development and cardiovascular abnormalities. This study will help to modulate the therapeutic strategies of hypertension.

## 1. Introduction

Hypertension is considered one of the most important and well-established causes of cardiovascular abnormalities, stokes, and premature mortality globally [1]. It has been reported that more than 1.39 billion individuals around the world are suffering from hypertension [2]. By the end of 2025, it is expected that the percentage of hypertensive patients will reach up to 60% of the total population. The hypertension prevalence among different regions may be explained by reasons such as improper diet, obesity, and lack of exercise [3]. Therefore, it is a priority for scientists to critically investigate the causes and processes involved in this disease to prevent the risks effectively [4].

DNA microarrays provide prevailing and efficient techniques for simultaneously exploring the expression patterns of thousands of different genes. A common use for microarrays is to study changes in gene expression under various experimental conditions of interest (e.g., cases vs. control). A common approach to data analysis is to use a set of specific important genes to predict biological or therapeutic outcomes. Pharmacogenomics is a vital approach for microarray gene expression profiles. A subset of the known genes will be used in the development of candidate genes or biomarkers. In drug development, the gene candidate is based on the patient’s genomic profile and helps to define how the benefits and side effects of a drug differ in the target patient population based on the germline of the patient and the genomic attributes of the affected tissue. By identifying groups of patients who are likely to benefit from the therapeutic effect and avoiding serious adverse events, the drug therapeutic index can be drastically enhanced. Gene selection is considered the first step in development. The universal principle of gene selection is to identify the genes that explain the underlying mechanism of disease progression, known as disease marker genes, their pathways or modes of action, and known interpretations. Choosing a subset of genes from the original thousands of genes increases the computational challenges [5,6]. The genetic basis of hypertension is gaining attention and the variations in alleles, gene expression, and protein level changes are important genomic factors [7,8]. Several studies have proven the role of differentially expressed genes in disease development. Most of the genes involved in hypertension have been reported as potential therapeutic drug targets. WNK kinases and angiotensin-converting enzyme 2 expression levels influence the development of hypertension in humans. Recent reports have shown significant variations in endothelin 1 (ET)-1 during cardiovascular diseases. It has been observed that higher levels of ET-1 are found in the plasma samples of hypertensive individuals. ET-1 also contributes to pulmonary vascular resistance [9]. Based on genomic transcriptomic level studies validated by qPCR, it has been identified that the differential expressed genes TcTex1, Myadm, Lisch7, Axl-like, Fah, PRC1, and Serpinh1 are associated with hypertension [10]. The renin–angiotensin–aldosterone system (RAAS) maintains blood pressure and hemostasis in the human body [11]. The genes influencing the RAAS ultimately affect blood pressure, including angiotensin-converting enzyme (ACE), angiotensin-1 (AGT), angiotensin-II type 1 receptor (AGTR1), and aldosterone synthase (CYP11B2) genes [12]. These genes are key targets in the hypertension therapeutic strategy, which increases blood pressure through arteriolar vasoconstriction, salt and water retention, and cardiac remolding or hypertrophy [13]. Quantitative real-time PCR (RT qPCR) is considered as the method of choice in the profiling of gene expression (mRNA levels) and follow-up validation with extended dynamic range and sensitivity based on its high accuracy rate [14,15]. The 2-ΔΔCT method is a useful approach to investigate the relative variations in gene expression from qPCR experiments [16].

In this study, we aimed to identify the possible candidate genes of hypertension followed by experimental validation. The system-level analysis screened out the key genes from cDNA datasets. Based on the robust multi-array analysis and differential studies, the functional-level associations of hypertension-related DEGs were studied. The qPCR analysis verified the dysregulation of these genes and their pathological role in hypertension. These findings will help to understand the genetic basis of the disease and will modulate the therapeutic strategies against hypertension.

## 2. Results

### 2.1. Normalization, Meta-Analysis, and Cross-Validation of Gene Expression Data

**Figure 1:**
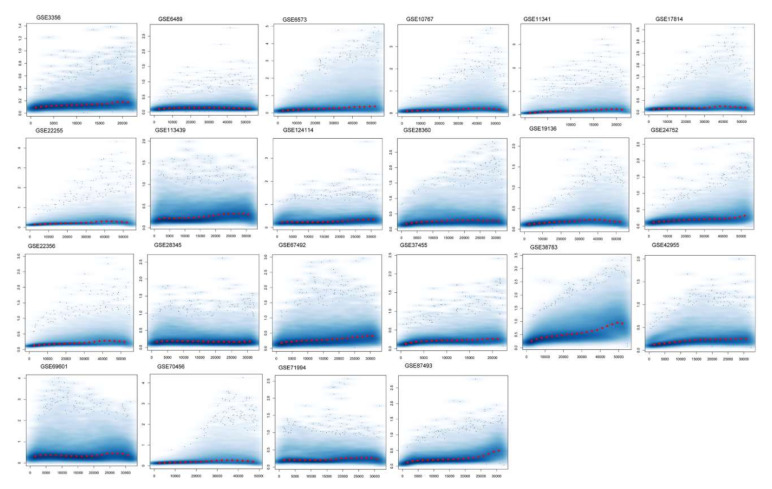
Normalization of differentially expressed genes. The figure shows a density plot of the standard deviation of the intensities across arrays on the y-axis versus the rank of their mean on the x-axis. The red dots, connected by lines, show the running median of the standard deviation. After normalization and transformation to a logarithm(-like) scale, one typically expects the red line to be approximately horizontal; that is, to show no substantial trend. In some cases, a hump on the right hand of the x-axis can be observed, which is symptomatic of the saturation of the intensities.

We used a publicly available human cDNA dataset to investigate the differentially expressed values of hypertension. We retrieved 22 Affymetrix cDNA datasets of hypertension for differential expression analysis. Each dataset has a different number of samples and genes derived through mRNA expression profiling using different Affymetrix platforms for hypertension. The data were normalized and missing values were imputed. The normalized distance between the array of DNA chips and the individual arrays of each dataset for the median expression level indicates the quality of the arrays. The gene–gene covariance matrix across all arrays in each dataset when ignoring missing values was computed to check sure whether they were on the same scale and to log-transform the arrays. The quantile normalization of the probes showed a quality histogram indicating normalized intensity between arrays of the entire DNA chip. The patterns in this smoothed histogram revealed the distribution of the arrays, having similar shapes and ranges (Figure 1). A meta-analysis of differential expression showed 07 DEGs of hypertension at *p* < 0.05. These genes presented directional uniformity in the cohorts under study. All DEGs showed correlations with hypertension. ADM, EDN1, ANGPTL4, NFIL3, MSR1, CEBPD, and USP8, based on their FDR values (Table 1) (<0.05, *p*-values (≤0.05)), were the top DEGs for hypertension (Supplementary Table S1).

We excluded any subgroup without repetition from comparisons of accuracy and verification of differential analysis, and the generalized linear model’ ‘cv.glm’ method measured the error of the cross-validation prediction. The Gaussian dispersion criterion was 0.00509, indicating the degree of confidence (Table 2). With K-fold estimation we obtained the same delta value of 0.00501, as we used the LOOCV approach (during raw cross-validation and afterward during modified cross-validation). The substantial codes (0.1, 0.01, 0.001, and 0.05) with residuals of limited deviance suggested the consistency of the differential analysis. To verify the dataset reliability for identification of variation at the transcriptional level in the original samples, the normalization process was used to standardize sample handling techniques and to assess the optimal RNA variability threshold using discrimination measures for statistical and algorithmic analyses.

### 2.2. Gene Ontology and Pathway Enrichment Analyses

**Figure 2:**
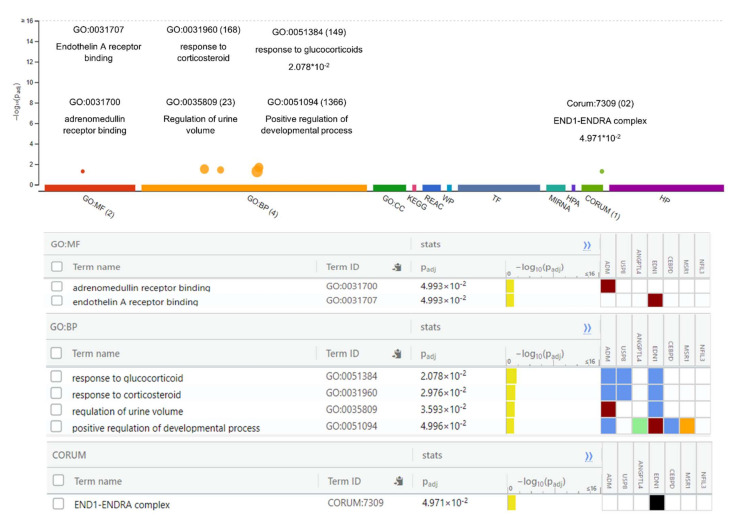
Gene Ontology (GO) analysis of differentially expressed genes. The GO analysis indicates important molecular functions and includes the top-ranked GO categories classified according to the levels of differentially expressed genes enriched in the three major classifications.

**Figure 3:**
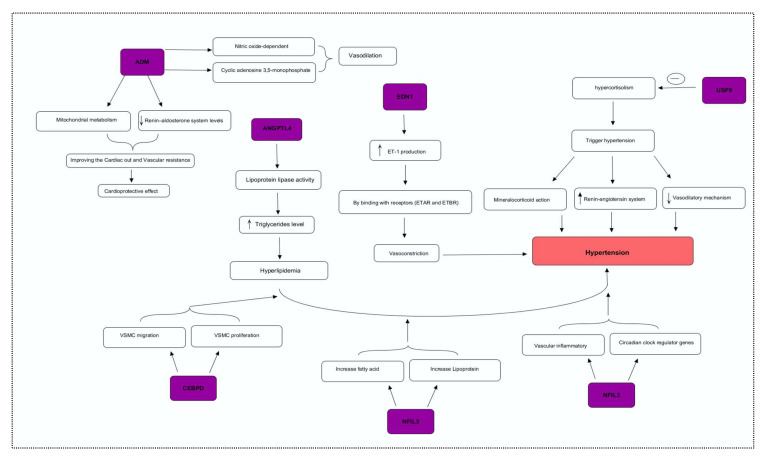
Integrative genome pathway remodeling study was used to map the possible mechanisms of the DEGs.

The Gene Ontology analysis of DEGs showed significantly enriched terms. These genes showed enrichment significantly linked to endothelin A receptor binding, adrenomedullin binding, regulation of urine volume, responses to corticosteroids and glucocorticoids, and positive regulation of the developmental process (*p*-value < 0.05). The function of the gene and its regulation, subtypes, and cellular processes play key roles in understanding its biology and the dysregulation of these biological processes causing hypertension and other cardiovascular diseases (CVS) (Figure 2). The genes were further examined to estimate the molecular mechanism involved in hypertension. The pathway enrichment analysis revealed the roles of the HIF-1-α transcription; endothelin; GPCR-binding ligand; and signaling pathways of EGF, PIk3, Arf6, S1P1, and PGDF in hypertension (Table 3). The network created after reconstruction demonstrated that a number of pathways participated in the pathogenesis of hypertension. This analysis highlighted the genes and pathways in the molecular and cellular functions and other signaling components of CVS (Figure 3).

### 2.3. Transcription and Motif Analysis

**Figure 4:**
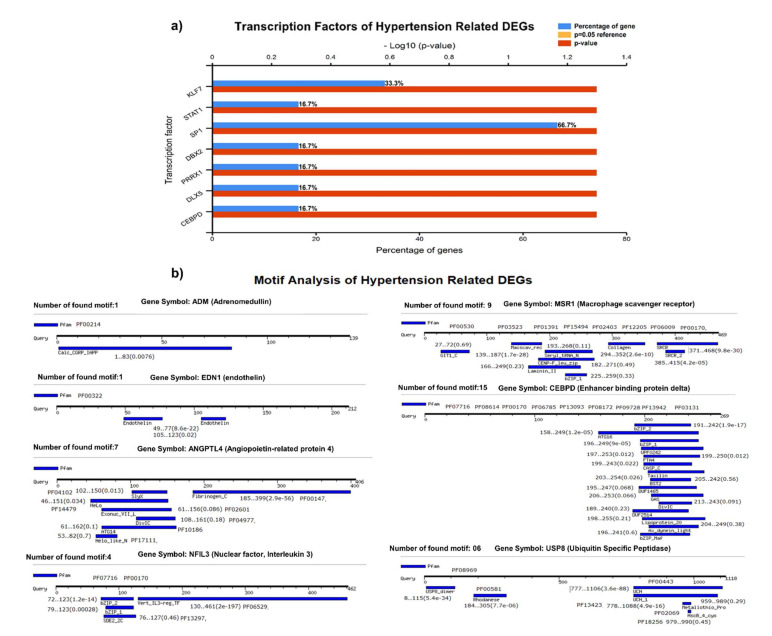
(**A**) Transcriptional factors of hypertension-related DEGS, identifying SP1, KLF7, STAT1, DBX2, PRRX1, and other regulatory factors. (**B**) Motif analysis highlighting a significant number of functional motifs associated with important biological functions.

We identified the transcriptional factors of DEGs with substantial *p*-values (<0.05), including SP1 (66.7%), KLF7 (33.3%), STAT1, DBX2, PRRX1, DLX5, and CEBPD (16.7%), (Figure 4A). We observed a greater number of motifs (15 motifs) in enhancer-binding protein (CEBPD), followed by 9 motifs in macrophage scavenger receptor (MSR1). The motifs were scanned with a significant cutoff value of <0.0002 under default parameters (Figure 4B).

### 2.4. Mutation Analysis

**Figure 5:**
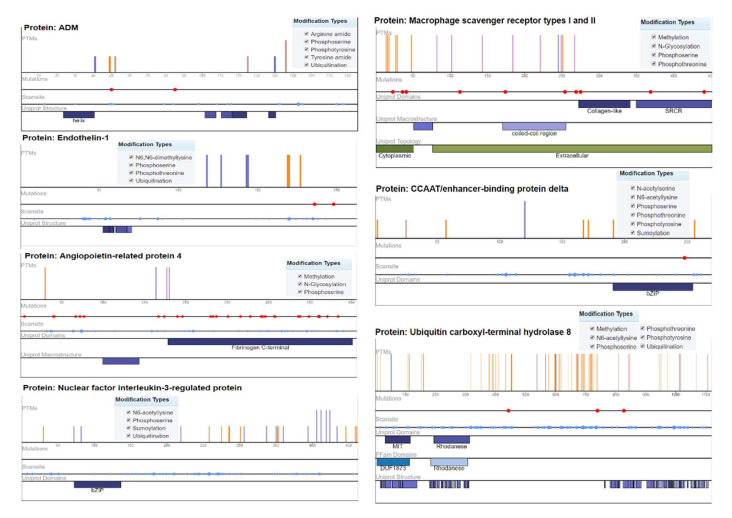
Mutation analysis of hypertension-related DEGs indicating post-translational modifications with significant cutoff parameters. It highlights the significant disordered regions of the proteins with a pathophysiological role in disease development.

Ubiquitin carboxyl–terminal hydrolase 8 (USP8) has six modification types, including phosphothreonine methylation, phosphotyrosine, phosphoserine, N6-acetyllysine, and ubiquitination, with recurrent mutations of 57 modified residues. The mutation visualization plot shows that USP8 has a direct network-rewiring mutation and that the modified amino acids Y, R, S, K, and T appeared throughout the various sites of the protein sequence, indicating a significant disordered region (57%). Similarly, CCAAT/enhancer-binding protein delta protein (CEBPD) has six phosphothreonine N-acetyl serine, phosphotyrosine, phosphoserine, N6-acetyllysine, and SUMOylation modification types, with the modified amino acids of Y, K, S, and T. The protein encoded by this intron gene is a bZIP transcription factor that can bind as a homodimer to certain DNA regulatory regions. There are 8 modified residues predicting that 8% of the sequence is disordered. Adrenomedullin (ADM) is a 52 AA peptide with several functions, including vasodilation, regulation of hormone secretion, and promotion of angiogenesis, indicating that 45.41% of the sequence is predicted to be disordered. This protein has 5 modification types, including phosphoserine, tyrosine amide, arginine amide, ubiquitination, and phosphotyrosine. The angiopoietin-related protein 4 (ANGPTL4) isoform is a precursor that encodes a glycosylated, secreted protein containing a C-terminal fibrinogen domain. The encoded protein is induced by peroxisome proliferation activators and functions as a serum hormone that regulates glucose homeostasis, lipid metabolism, and insulin sensitivity. Here, 16.75% of the sequence is predicted to be disordered with PTM types of phosphoserine, N-glycosylation, and methylation (Figure 5).

### 2.5. Protein Product Co-Expression Network Analysis

**Figure 6:**
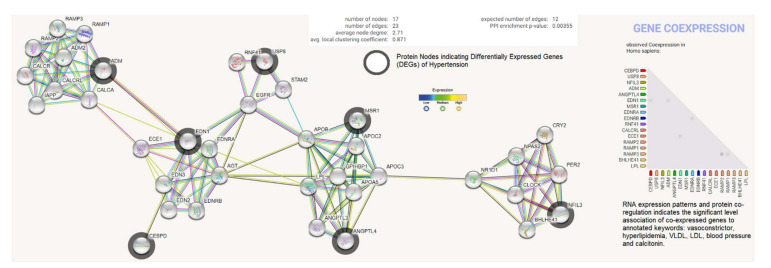
Protein product co-expression network analysis using STRING database version 11.0. The protein network was calculated based on the neighborhood score with higher confidence (confidence score > 0.99). Nodes represent proteins and edges indicate interactions. The co-expression scores based on RNA expression patterns and protein co-regulation were studied through the STRING database and annotated keywords (FDR < 0.05) were observed.

The differentially expressed genes (ADM, EDN1, ANGPTL4, NFIL3, MSR1, CEBPD, and USP8) were investigated for possible interactions with each other using STRING biological data. It was posited that the most differentially expressed genes would have strong interactions with each other. The protein–protein interaction (PPI) network contains 17 nodes (each node indicates proteins) and 23 edges (present interaction). The protein network indicates the enriched co-expressed genes (PPI enrichment *p*-value: 0.00355) functionally associated with circadian rhythm, cholesterol metabolism, vascular smooth muscle contraction, the PPAR signaling pathway, relaxin, and the calcium signaling pathway. We obtained the Pfam protein domains, namely calcitonin/CGRP/IAPP family, endothelin receptor activity-modifying family, PAS domain, PAS fold, and hormone receptor domain (FDR < 0.05), which were mostly associated with the differentially expressed genes. Based on the protein product co-expression data, we analyzed the expression levels of CEBPD, MSR1, and NFIL3 genes and observed that these were downregulated. The RNA expression pattern and protein co-regulation results indicate the significant association levels of co-expressed genes with annotated keywords, namely vasoconstrictor, hyperlipidemia, VLDL, LDL, blood pressure, HDL, lipid metabolism, disease mutation, and calcitonin (Figure 6).

### 2.6. Clinical Description of Samples

Out of the selected patients, 28 individuals were males and 22 were females in each control and case. For hypertensive patients, the BMI values of 22 individuals were recorded within the range of 25–30 kg/m^2^, indicating overweight, while 22 were obese as compared to the control. The mean systolic and diastolic BP values of hypertensive patients were 152.2 ± 11.2 mmHg and 94.9 ± 4.23 mmHg, respectively, compared to control, while the mean systolic and diastolic BP values were 125.7 ± 5.32 mmHg and 82.8 ± 2.8 mmHg, respectively (Table 4).

### 2.7. Validation of qRT-PCR Assay and Expression Profiling

**Figure 7:**
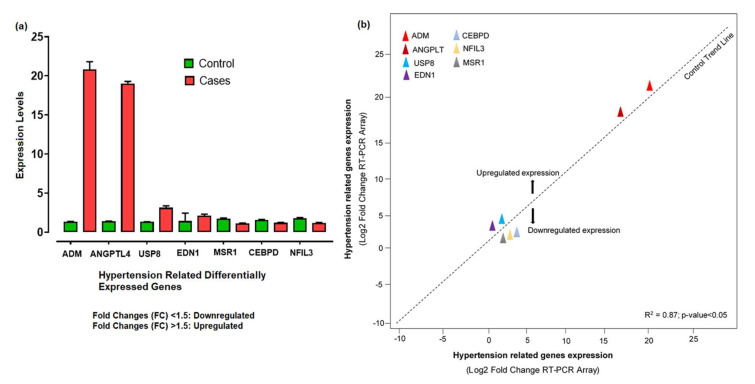
(**a**) Aberrant expression levels of differentially expressed genes in hypertension cases and controls based on the fold changes of gene expression. (**b**) The qRT-PCR array validation of the differentially expressed genes. The plot graph shows the correlation between the expression levels of hypertension-related DEGs measured by array analysis and expression levels measured by individual qRT-PCR. The −ΔΔCt method was applied for this analysis.

The 260/280 ratios between 1.5 and 2.7 indicated the high-quality RNA, while the quantity range for our samples was about 800 to 1250 ng/μL. The RNA quantification showed a significant level for further cDNA synthesis. The relative expression levels of 7 hypertension-related differentially expressed genes shown by qPCR were analyzed and calculated according to the relative expression quantity 2−ΔCT formula, where ΔCT = CT value of target gene–CT value of the internal reference gene (GAPDH). CT values were generated from the absolute quantification, indicating the quality of the results and providing information on the actual levels. The probe for the RT-PCR was constant enough, as no enhanced fluorescence signal was seen after the reaction. The relative expression levels showed that ADM, ANGPTL4, USP8, and EDN1 were upregulated, with significant fold changes (2^−ΔΔCt^) compared to control. The substantial aberrant expression of ADM and ANGPTL4 correlated with disease development and progression. However, CEBPD, MSR1, and NFIL3 were downregulated (Figure 7a). Based on the −ΔΔCt method, we observed a significant level (R^2^ = 0.87; *p*-value < 0.05) of correlation between the expression levels of hypertension-related DEGs measured by array analysis and expression levels measured by individual qRT-PCR (Figure 7b).

**Figure 8:**
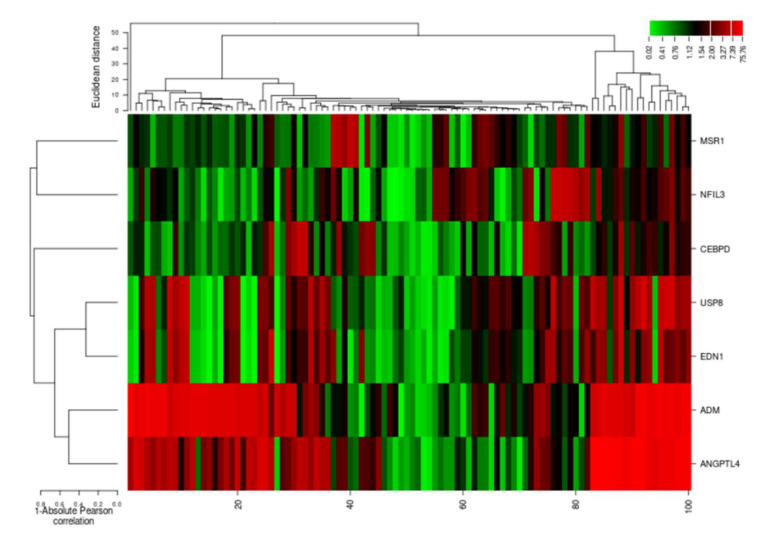
Hierarchical cluster analysis heatmap indicating expression profiles of differentially expressed genes (ADM, ANGPTL4, USP8, EDN1, NFIL3, MSR1, and CEBPD). The columns in the figure represent the samples and the rows represent the differentially expressed genes.

The cluster analysis indicated the gene expression profiles of the two groups (cases and controls), with significant expression level differences. The columns represent samples and the rows represent differentially expressed genes. Figure 7 shows that most of the samples have differential expression profiles compared to the left-hand dendrogram, where some genes have similar expression patterns (Figure 8). These genes may have similar functions or participate in the same biological process. Genes with the highest differential expression were named as a gene cluster. We observed significant gene clusters, including ADM, ANGPTL4, and USP8 expressed differentially in a number of samples (fold change ≥2 and *p*-value < 0.05).

## 3. Discussion

Hypertension risk factors are commonly reported in developing countries. The incidence of hypertension is frequently rising, but its control is inadequate worldwide [17,18]. Gene expression in humans has proven significant for identifying genetic determinants of phenotypic traits and for pinpointing genes related to complex traits. The most common diseases found in humans are due to the interactions of many genes; therefore, a more consolidative approach of biology is needed to resolve the intricacies and reasons behind such diseases [19]. In this study, we used various approaches by utilizing the system-level framework in the meta-analysis of the cDNA Affymetrix dataset in CELL format presented publicly on NCBI to extract the most common genes involved in hypertension. The advancement in the microarray analysis empowered scientists to investigate a large number of genes at once and to find genetic evidence for various diseases [20].

The gene ontology and pathway enrichment analyses showed critical associated pathophysiological development in hypertension. We found the pathways such as HIF-1-α transcription; endothelin; GPCR-binding ligand; and signaling pathways of EGF, PIk3, Arf6, S1P1, and PGDF were involved in biological processes. HIF-1alpha is a transcriptional factor that acts in response to hypoxia. Studies reported that HIF-1alpha and ARF6 regulate the role of vascular endothelial growth factors in pulmonary arteries, showing a vital part of the pathogenesis of hypertension and hypoxic artery remodeling [21,22]. The endothelin production is enhanced during hypertension, which simulates inflammation and vessel constriction. Therefore, the blockage of endothelin was an effective and promising target in the management of hypertension [23]. GPCR ligands are still considered a relevant drug target in CVS disease, as a number of GPCR-binding drugs with sympathomimetic effect cause vasoconstriction and smooth muscle proliferation of pulmonary arteries [24]. PIK3 contributes to the vascular response via its action on the L-type calcium, which affects the regulation of blood pressure [25]. Age was found to be a critical hazard calculator for hypertension. As the age increased, the predominance of hypertension increased among both genders. Comparative findings were reported by other studies, showing that progressing age was positively related to hypertension [26]. In our study, we found that at ages over 50, more hypertension cases appear (31 subjects). With advancing age, the aorta and coronary artery wall will stiffen, and this contributes to hypertension [26]. Out of 50 cases of hypertension, 28 were males and 22 were females. Different studies have suggested a higher prevalence rate of hypertension in males than females [26,27]. One of the conceivable reasons for this gender dissimilarity in hypertension predominance could be due to natural sex distinctions, while behavioral hazard components such as smoking and alcohol consumption may also be more common in males. However, females are more curious about their wellbeing, so they are more likely to have better health [28]. Similarly, BMI is another major risk factor involved in hypertension progression, as also reported in other studies [29,30]. We found that the percentage of overweight and obese subjects was greater in hypertensive samples than in the control.

Real-time (RT) PCR analyses were used to confirm the results of the microarray datasets, which demonstrate the true changes. We identified the potential genes, including ADM, EDN1, ANGPTL4, NFIL3, MSR1, CEBPD, and USP8 (*p* < 0.05), via differential analysis (DEGs). Furthermore, we investigated these DEGs for the expression level changes in our population and observed the findings in cases and controls. A significant fold variation was observed during the RT-PCR analysis in the target samples using the 2^−ΔΔCt^ method [31]. The real-time PCR of the ADM gene demonstrated 20.83 times greater gene expression in target samples compared to control. Hypertension is regulated mainly by the autonomic nervous system, the renin–angiotensin system, and nitric oxide (NO) [32]. Recent studies suggested the role of neurohumoral factors in the development of hypertension. Adrenomedullin (ADM) can be produced by numerous tissues, including the myocardium, adrenal medulla, and central nervous system, and has various pathophysiological functions [33]. ADM shows prominent effects, including dilation of vessels, natriuresis, and NO production. The level of ADM plasma concentration was significantly higher in hypertensive patients, representing its protective and therapeutic role in cardiovascular abnormalities [34,35]. The role of the ADM gene mechanism was found in clinical research related to hypertension. The findings suggested that ADM showed vasodilator effects mediated via cyclic adenosine 3,5-monophosphate and nitric-oxide-dependent mechanisms [36]. The cardioprotective role of ADM against cell death in heart diseases is through disruption of mitochondrial metabolism and by reducing renin–aldosterone system levels, thereby improving cardiac output and vascular smooth muscle resistance [37]. Similarly, the ANGPTL4 gene was found to be upregulated compared to control, indicating a substantial level of variation [38]. It has been shown that the ANGPT4 gene was involved in angiogenesis, leading to vascular disease [39,40]. Studies have confirmed the association of ANGPTL4 with lipid metabolism and it has been observed that the inhibition of this gene significantly decreases the triglyceride level, which ultimately reduces cardiac issues [41,42]. ANGPTL4 is highly expressed in the endothelial cells, leading to hyperlipidemia [43,44]. It has been reported that hyperlipidemia and hypertension are linked in many aspects, sharing certain potential common risk factors [45]. In some studies, the amounts of ANGPTL4 were increased in both plasma and adipose tissues of hypertensive samples as compared to control, highlighting its probable contribution and therapeutic worth in the mechanism of hypertension, having a role in the regulation of lipid metabolism by constraining the activity of lipoprotein lipase [40,46].

The absolute quantitative analysis showed that the USP8 gene is another important gene, which is expressed with a fold difference 3 times higher than controls. Studies published in 2013 indicated the potential role of this gene in the trafficking of the sodium channel [47]. The higher sodium channel endocytosis might be the reason for hypertension [48]. USP8 is considered a novel therapeutic drug target in Cushing disease (CD). CD induced a series of complications such as hypertension, obesity, and diabetes mellitus [49]. Hypertension is notably associated with the length of hypercortisolism and outcomes from the interplay among several pathophysiological mechanisms. Glucocorticoids trigger hypertension through numerous mechanisms, including their core mineralocorticoid action, through renin–angiotensin system activation, by augmentation of vasoactive properties, and by inhibiting the vasodilatory mechanism [50]. Therefore, we proposed that USP8 target drug therapies will play a vital role in hypertension treatment. Endothelins-1 (EDN1) is present in abundant quantities in blood vessels and is a potent vasoconstrictor peptide [51]. In our hypertension cases, the EDN1 gene was found to be over-regulated compared to control. EDN1 induces vascular hypertrophy and endothelial dysfunction. Endothelin receptor blockers reduce blood pressure and vascular hypertrophic remodeling [52,53]. The possible reported mechanism of the EDN1 genes in hypertension is that the higher transcription of EDN1 increases the ET-1 production by the endothelial cells in the blood vessels, causing powerful constriction in the vascular smooth muscle cells and increasing peripheral resistance mediated via smooth vascular muscle subtype receptors (ET_A_R and ET_B_R) during pathological conditions [54]. Therefore, EDN1 is considered a potential therapeutic drug target in hypertension [55]. Nuclear factor, interleukin-3-regulated (NFIL3) was constantly expressed at a high level with the low-sodium diet, although the expression became downregulated along with the progression in the sodium-loaded diet [56], and parallel findings were observed in our study [57]. Circadian rhythms influence transcriptional levels, affecting cell metabolic pathways. Alterations in circadian rhythm increase the risk of hypertension. Recently, another possible method of hypertension was proposed, namely perivascular inflammation, which participates in the vascular inflammatory response [58]. NFIL3 could be a possible target, as it is a circadian clock regulator gene that might be involved in hypertension and lipid metabolism [59,60]. We observed that macrophage scavenger receptor 1 (MSR1) and CEBPD are downregulated in hypertension cases compared to control [61]. MSR1genetic polymorphisms are significantly associated with hypertension [62]. The expression of the MSR1 gene in atherosclerotic vessels increases the accumulation of fatty acids and lipoproteins in the blood, which in turn causes hypertension and ultimately damages the basement membrane of arteries [63]. Furthermore, MSR1 is considered a potential marker in hypertension, in addition to other traditional risk factors [62]. CEBPD encodes transcription factors such as CCAAT and platelet-derived growth factor-α receptor (PDGF-αR) expression, causing vascular smooth muscle cell proliferation and migration, resulting in genetic hypertension [64]. Moreover, CEBPD has a facilitatory role in binding with other transcription factors and contributes to the vibrant alteration of the chromatin architecture, with recognized effects in hypertension [65,66]. Based on its significant role, CEBPD is considered a molecular marker of blood pressure [67]. These findings demonstrate that the dysregulated expression of selective genes is correlated with the pathophysiological role of hypertension.

## 4. Materials and Methods

### 4.1. Normalization and Differential Expression Analysis

We retrieved publicly available microarray datasets from the Gene Expression Omnibus (GEO) database in a compatible (CEL) format using the key term “hypertension” to download related datasets accessible until 1 June 2019, based on the following parameters: (i) tissue specificity; (ii) sample size; (iii) array size and pattern: 712 × 712, 1050 × 1050 and 1164 × 1164; (iv) platform, i.e., “Affymetrix U133Plus2.0”, annotation probe “HGU133plus2” (Table 5).

Various Bioconductor packages with the R platform were used (Affy, AffyQCReport, AffyRNADegradation, AnnotationDbi, Annotate, Biobase, Lima, and HGU133a2cdf) to assess the quantifiable outcomes. Robust multi-array analyses (RMA) were used to quantify perfect matches (PM) and mismatches (MM). For the RNA degradation analysis of the samples, AffyRNAdeg, summary AffyRNAdeg, and plotAffyRNAdeg were used to check the quality of RNA.

Normalization was used to compare microarray datasets. The pheno-data files of these datasets were organized in an identifiable format [68]. Background correction, i.e., for a perfect match (*PM*) and mismatch (*MM*), was calculated as given in the equation. Robust multi-array analysis (RMA) was used to remove local artifacts and noise [69,70]:

*PMijk* = *BGijk *+* Sijk* (1)

where *PM* is a perfect match with the background (*BG*) caused by optical noise and nonspecific binding (*S*); *ijk* is the signal for probe *j* of probe set *k* on array *i*.

*BG*(*PMijk*) = *E*[*Sijk*|*PMijk*] > 0 (2)

*Sijk*∼*Exp*(*λijk*) *BGijk*∼*N*(*βi*,*σ*^2^) (3)

Here, PM-data combines a background (*BG*) and a signal or expression (*E*). The Bioconductor “Array Quality Metrics” package was used to analyze the dataset, which was normalized to the median expression level of each gene [69,71,72]. The expression value of a transcript with a *p*-value < 0.05 was considered a marginal log transformation and the quantile normalization of the arrays brought them to the same scale. The gene–gene covariance matrix of each dataset, ignoring the missing values, was calculated across all arrays (54,675 affyIDs). The formula for the transformation was:

*Xnorm* = *F*2^−1^(*F*1(*x*)) (4)

where *F*1 and *F*2 are the distribution functions of the actual and reference chips, respectively.

We used the RMA algorithm to calculate the averages between probes in a probe set to obtain a summary of intensities. The Bioconductor “AffyRNADegradation” package was used for the RNA degradation analysis and to measure the quality of RNA in these samples [73,74]. In this study, we identified differentially expressed genes of each dataset by pairwise comparison [75] by selecting two tissues or cell types (indicating cases vs. control). After a baseline and normalized median expression, significant DEGs were selected based on the following threshold parameters: FDR < 0.05, logFC > 1, *p*-value ≤ 0.05, and average expression level (AEL) ≥ 40%. The list of genes was further studied for significant overlap with various gene sets for functional annotation.

### 4.2. Meta-Analysis

We performed a meta-analysis of the twenty-two Affymetrix cDNA datasets (cohorts) related to hypertension. We conducted this analysis using random effects models of R package Meta (http://cran.r-project.org/web/packages/meta/index.html, accessed on 14 November 2021). For the *p*-value significance (*p* < 0.05), Cochran’s Q statistic was used to test the heterogeneity of each gene. We used the Benjamini–Hochberg method to calculate the FDR for differentially expressed genes in relation to hypertension following the meta-analysis. The Benjamini–Hochberg method was used for multiple testing corrections [76]. DEGs and duplicated pots along with the measurements of quality weights were shortlisted through the Limma package, a modified statistic method. The moderated statistics were calculated; genes were ranked, and *p*-values were measured [77].

### 4.3. K-Fold Cross-Validation

We used K-fold cross-validation and bootstrap tests to evaluate the accuracy in differential analysis. This approach has the advantage of ultimately using all data samples for training and research [78]. This was used to estimate the simulation analysis and to equate and choose the right model for predictive modeling errors. Usually, this method makes it is easier to calculate the estimated average error and is used to validate the differentiated genes with the Bioconductor Boot package. In molecular analysis, boot trapping is effectively used to correct the bias. The generalized Gaussian linear models were applied and the ‘cv.glm’ method was employed to test the k-fold cross-validation. It approximates the true error as the average error:

(5) $$E=1/K\sum_{i=K}^{K}Ei$$

The Gaussian rule remains in leave-one-out cross-validation (LOOCV). The LOOCV approach is called the test set and the remainder of the data are used as a training set [79]. For training and other testing, we used N-1 subsets. Increasing the number of plugs would reduce and make valid the distraction of the true error rate estimator [80]. As the average error rate in test cases, the true error is evaluated:

(6) $$E=1/N\sum_{i=K}^{N}Ei$$

### 4.4. Gene Ontology (GO) and Pathway Enrichment Analyses

To identify the functional genes and biological pathways significantly involved in selected DEGs, Gene Ontology analyses were performed using the g:Profiler online server, representing gene product properties and biological functions [81]. GO is a standard classification system used to determine the significant signaling pathways for biological and molecular functions and cellular components of differential expressed genes. We analyzed the pathway enrichment of DEGs using the FunRich tool version 3.1.3 with significant *p*-values < 0.05 [82]. PathVisio3tool was utilized to find and reconstruct the cellular pathways of potential candidate genes and possible mechanisms of DEGs were studied based on the available clinical research literature and databases [83].

### 4.5. Examination of Transcription and Regulatory Motifs of DEGs

We predicted the potential regulators of differentially expressed genes using the FunRich tool, as cells evolved a related transcription network composed of transcription factors (TFs) and other signaling molecules. These gene regulators are associated with various biological and pathological functions [84]. In proteins, the motive defines the connection between the secondary structural elements, and in all instances the spatial sequences of residues of the amino acids, encoded by genes in any order, may be similar. We used the Motif Search online tool to analyze the link between the primary sequence and the tertiary structure for the structural motives of differentially expressed genes (https://www.genome.jp/tools/motif/, accessed on 14 November 2021).

### 4.6. Mutation Analysis

Mutations resulting from a pathological, environmental, inherited disease process and other related conditions can be understood to decode genetic variations by genotype–phenotype associations. The human genome contains thousands of SNVs (single-nucleotide variants), and many are known for the progression of the disease. Approximately 21 percent of amino acid substitutions are known to be associated with disease progression in correspondence with missense single-nucleotide variants located at PTM (post-translation modification) protein sites. The chemical modification of the amino acid, thus, extends the functionality of the associated protein [85]. Mutations of differentially expressed genes were analyzed using the online ProteomeScout Portal [78]. The needle plot mutation analysis provided a visual overview of the position, frequency, and functional significance of all identified mutations in our DEGs. PTM sites with the types of mutations and the predicted disordered region of protein sequences were observed.

### 4.7. Protein Product Co-Expression Network Analysis

To investigate the interactions between the protein products of the top-ranked differentially expressed genes related to hypertension, STRING database version 11.0 (https://string-db.org/, accessed on 14 November 2021) was used to construct a protein co-expression network with nodes consisting of genes and edges derived from experimentally validated protein–protein interactions. The protein network calculated based on the neighborhood score (*nscore*) was computed from the interactive nucleotide count with higher confidence and true positive values. The co-expression score based on RNA expression patterns and protein co-regulation was studied using the STRING database, and annotated keywords (FDR < 0.05) were observed.

### 4.8. Ethical Approval, Collection of Blood Samples, and Clinical Description

The approval of the study and informed consent were obtained from the Research Ethical Committee, Riphah Institute of Pharmaceutical Sciences (Ref. No. REC/RIPS/2017/015). We collected a total of 100 individual blood samples with an equal ratio of controls to cases (*n* = 50). Blood samples of hypertension patients were collected randomly from Khyber Pakhtunkhwa, a Province of Pakistan. The blood pressure (BP) values of selected participants were checked twice on the resting phase using a digital automatic electronic monitor (OMRON M2 basic) before sampling. The BMIs of the selected individuals were calculated as per WHO-approved guidelines [86].

#### 4.8.1. Inclusion Criteria

Inclusion criteria were as follows: (1) pathologically confirmed cases of hypertension; (2) new patients diagnosed by the Khyber Pakhtunkhwa Hospital for the first time; (3) patients aged ≥18 years; (4) a random sampling technique was used to collect blood samples for each case and control from different individuals and prior consent from subjects of some families (especially for females). Patients and their family members agreed to provide blood samples for scientific research and consent to the publication of research data.

#### 4.8.2. Exclusion Criteria

Exclusion criteria were as follows: (1) pathologically confirmed local vascular invasion; (2) cases with multiple and complex diseases; (3) cases with diabetes, cancer, or immune disorders; (4) patients with a history of surgery in the past 3-years.

### 4.9. RNA Extraction and Quantification

Here, 300 µL of each blood sample was transferred into a 1.5ml Eppendorf tube containing 700 µL triazole. These tubes were gently mixed and incubated at 25 °C for 5 min. Next, 400 µL of chloroform was added into these tubes and kept for 3 min. The mixture was centrifuged at 12,000 rpm for 10 min at 4 °C for phase separation. The aqueous upper layer was transferred into a new 1.5ml tube while keeping it on ice, then isopropanol was added in equal proportions. Tubes were kept on ice (−20 °C) for 10 min in a horizontal position to precipitate RNA. Samples were centrifuged at 12,000 rpm at 4 °C for 10 min, then the supernatant was discarded. The pellet was washed twice with 1 ml of 70% ethanol at 7500 rpm for 5 min and was air-dried. Next, 40 µL of RNase-free water was added and RNA samples were stored at −80 °C [79,80]. RNA was quantified at 260, 280, and 320 nm by Nanodrop (Skanit RE 4.1, Thermo Scientific, Waltham, MA, USA) [87].

#### 4.10. cDNA Synthesis

The extracted RNA was converted into cDNA using a cDNA synthesis kit (Vivantis cDSK01-050). Next, 10 µL of the cDNA synthesis mix was added to each RNA–primer mixture. After centrifugation at 10,000 rpm, the samples were incubated at 42 °C for 60 min. The reaction was terminated by incubating the tubes at 85 °C for 5 min. The tubes were chilled on ice and centrifuged under the same conditions. The synthesized cDNA was directly used for further analysis [1,88].

#### 4.11. Quantitative Real-Time PCR Analysis

The PrimerBank server was used to design primers of differentially expressed genes [89] (Table 6). Polymerase chain reactions were performed on a Galaxy XP Thermal Cycler (BIOER, PRC) [90]. To validate the differentially expressed genes of hypertension, qRT-PCR was performed using MIC-PCR (BioMolecular System) under recommended conditions [91]. We selected the 7 top-ranked genes during the system-level analysis and observed that their correlation with the etiology of hypertension was not well studied. Reactions were set up to a final volume of 10 µL using 2.6 µL of cDNA (1:10), 5 µL of SYBR Green Master Mix, and 0.4 µL of each gene-specific reverse and forward primer. The final compositions of the reaction mixture remained the same for differentially expressed genes and reference genes. The GAPDH was used as an internal reference and a two-step procedure was applied, while qPCR was used to detect the expression of GAPDH. The relative expression level of each differential gene in each blood sample was calculated with the GAPDH expression level as the “1” standard value. The cycling conditions included: 95 °C for 12 min, 40 cycles of 95 °C of 15 s, 57 °C for 20 s, and 72 °C for 20 s. A final extension step was carried out at 72 °C for 10 min [92]. The PCR products were analyzed via melting analysis graph [93]. In the PCR process, during each cycle, the specifically amplified products are doubled in exponential form. The CT (cycle threshold) is a logarithmic value converted to a relative quantity [94]. The average CT values were calculated for both the target and reference genes. In the next step, the ΔCt (delta threshold) values were calculated for the target and reference genes using this formula: ΔCt = CT value of the target–CT value of the reference gene.

The ΔΔCt (delta threshold) value indicates the differences between the expression levels of differentially expressed genes and the reference gene [95]. Finally, 2ΔΔCt values were calculated, presenting the fold differences in gene expression of DEGs with controls [96]. We estimated the absolute correlation of these DEGs to allow a complete description of the expression profiles. The hierarchical clustering analysis of genes regarding their expression was evaluated [97] using an online one-matrix CIMminer tool [98].

## 5. Conclusions

This study helps to provide new insights into the discovery of new gene variants related to hypertension. Here, we found the significant association of ADM, EDN1, ANGPTL4, USP8, NFIL3, MSR1, and CEBPD genes with hypertension. The qPCR analysis and expression profiling highlighted the differential expression levels of these genes in cases compared to controls, revealing their pathological role in disease development. These molecular entities would be considered as potential drug targets that would help to modify the therapeutic strategies of hypertension and other cardiovascular diseases.

## Figures and Tables

**Table 1:** Common and related differentially expressed genes of each microarray dataset in hypertension.

Probe ID	Gene Symbol	Uniport ID	Protein Name
203973_s_at	CEBPD	CEBPD_HUMAN	CCAAT/enhancer-binding protein delta (CEBPD)
222802_at	EDN1	EDN1_HUMAN	Endothelin 1(EDN1)
203574_at	NFIL3	NFIL3_HUMAN	Nuclear factor, interleukin-3-regulated (NFIL3)
221009_s_at	ANGPTL4	ANGL4_HUMAN	Angiopoietin-related protein 4 (ANGPTL4)
202912_at	ADM	ADML_HUMAN	Adrenomedullin (ADM)
208423_s_at	MSR1	MSRE_HUMAN	Macrophage scavenger receptor 1(MSR1)
202745_at	USP8	H0YM17_HUMAN	Ubiquitin-specific peptidase 8(USP8)

**Table 2:** K-fold cross-validation using the Bioconductor “Boot” package based on Gaussian dispersion parameters.

	Estimate	Std. Error	t. Value	Pr (>|t|)
(Intercept)	0.000116	0.000312	3.99	<1.00 × 10^−11^ ***
x1	0.040024	0.001702	19.018	<1.00 × 10^−10^ ***
x2	−0.01042	0.001105	−4.017	<1.96 × 10^−9^ ***
x3	0.120113	0.003201	27.015	<1.00 × 10^−10^ ***
x4	0.210420	0.001412	20.200	<1.00 × 10^−12^ ***
x5	0.026013	0.002140	29.003	<1.00 × 10^−13^ ***
x6	0.231420	0.003263	25.012	<1.00 × 10^−11^ ***
x7	−0.01601	0.001561	−27.112	<1.00 × 10^−9^ ***
x8	0.001412	0.002211	19.115	<1.00 × 10^−11^ ***
x9	0.102122	0.003602	61.0716	<1.00 × 10^−13^ ***
x10	0.010010	0.000511	4.001	<1.00 × 10^−11^ ***
x11	0.030821	0.001403	21.003	<1.00 × 10^−10^ ***
x12	−0.01109	0.002014	−2.014	0.0078 *
x13	−0.14522	0.002919	−49.023	<1.00 × 10^−8^ ***
x14	0.010051	0.001240	1.312	5.28 × 10^−9^ ***
x15	−0.017581	0.001200	−18.102	<1.00 × 10^−10^ ***

Signif. codes: 0 ‘***’ 0.001 ‘**’ 0.01 ‘*’ 0.05 ‘.’ 0.1; number of Fisher scoring iterations: 2; $K: [1] 10; $delta: [1] 0.00501 = 0.00509; null deviance: 101602.1 with 49103 degrees of freedom; residual deviance: 2504.1 with 40119 degrees of freedom.

**Table 3:** Pathway enrichment analysis of DEGs using FunRich tools, showing enriched pathways associated with hypertension.

Pathway	Description	Count	Strength	False Discovery Rate
hsa04710	Circadian rhythm	6 of 30	2.02	3.75 × 10^−9^
hsa04979	Cholesterol metabolism	6 of 48	1.82	2.43 × 10^−8^
hsa03320	PPAR signaling pathway	4 of 72	1.47	0.00021
hsa04270	Vascular smooth muscle contraction	5 of 119	1.35	7.85 × 10^−5^
hsa04926	Relaxin signaling pathway	3 of 130	1.09	0.0200
hsa04020	Calcium signaling pathway	3 of 179	0.95	0.0412
hsa04144	Endocytosis	4 of 242	0.94	0.0157
hsa04080	Neuroactive ligand receptor interaction	4 of 272	0.89	0.0200
**Reactome pathways**				
**Pathway**	**Description**	**Count**	**Strength**	**False discovery rate**
hsa400253	Circadian clock	3 of 8	2.6	4.55 × 10^−6^
hsa1368108	BMAL1: activates circadian gene expression	3 of 11	2.16	6.66 × 10^−6^
hsa1227986	Signaling by ERBB2	3 of 22	1.86	3.11 × 10^−5^
hsa162582	Signal transduction	15 of 1358	0.77	8.60 × 10^−8^
**Annotated keywords (UniProt)**				
**Keywords**	**Description**	**Count**	**Strength**	**False discovery rate**
KW-0839	Vasoconstriction	4 of 5	2.63	3.59 × 10^−8^
KW-0162	Chylomicron	5 of 9	2.47	3.83 × 10^−9^
KW-0380	Hyperlipidemia	2 of 4	2.42	0.00037
KW-0850	VLDL	4 of 10	2.33	1.92 × 10^−7^
KW-0427	LDL	3 of 9	2.25	1.44 × 10^−5^
KW-0367	Hirschsprung disease	3 of 10	2.2	1.70 × 10^−5^
KW-0897	Waardenburg syndrome	2 of 7	2.18	0.00078
KW-0345	HDL	2 of 16	1.82	0.0028
KW-0730	Sialic acid	2 of 20	1.72	0.0038
KW-0027	Amidation	4 of 44	1.68	1.76 × 10^−5^
KW-0090	Biological rhythms	7 of 138	1.43	1.92 × 10^−7^
KW-0372	Hormones	4 of 87	1.39	0.00021
KW-0445	Lipid transport	4 of 110	1.28	0.00041
KW-0358	Heparin binding	3 of 87	1.26	0.0033
KW-0442	Lipid degradation	3 of 102	1.19	0.0047
KW-0165	cleavage on pair of basic residues	7 of 277	1.13	1.05 × 10^−5^
KW-0443	Lipid metabolism	6 of 447	0.85	0.0011
KW-0964	Secreted	6 of 1814	0.67	8.00 × 10^−7^
KW-0675	Receptor	11 of 1423	0.61	0.00034

**Table 4:** Clinical descriptions of individuals involved in the study.

Variables	Cases (*n* = 50)	Control (*n* = 50)
Age group		
18–65	50	50
Gender		
Male	28	28
Female	22	22
BMI (kg/m^2^)		
Normal	6	28
Overweight	22	16
Obese	22	6
Mean blood pressure		
Systolic BP (mean ± SD)	152.2 ± 6.02	125.7 ± 7.42
Diastolic BP mean ± SD)	94.9 ± 4.23	82.8 ± 3.65

**Table 5:** Phenotype characteristics data for cDNA datasets.

S. No.	Dataset Accession	AffyIDs	Total Samples	Size of Arrays	Tissues	Conditions
1	GSE6489	54675	6	1164 × 1164	Endothelial cells	Hypertensive vs. normatensive
2	GSE6573	54675	6	1164 × 1164	Adipose tissue	Hypertensive vs. normatensive
3	GSE10767	54675	7	1164 × 1164	Endothelial Cell	Hypertensive vs. normatensive
4	GSE17814	54675	18	1164 × 1164	Endothelial cells	Hypertensive vs. normatensive
5	GSE19136	54675	12	1164 × 1164	lLft mammary artery	Hypertensive vs. normatensive
6	GSE22255	54675	40	1164 × 1164	Blood cells	Hypertensive vs. normatensive
7	GSE22356	54675	38	1164 × 1164	Blood cells	Hypertensive vs. normatensive
8	GSE24752	54675	6	1164 × 1164	Blood cells	Hypertensive vs. normatensive
9	GSE37455	54675	41	1164 × 1164	Kidney	Hypertensive vs. normatensive
10	GSE38783	54675	24	1164 × 1164	Endothelial cell	Hypertensive vs. normatensive
11	GSE28345	32321	8	1050 × 1050	Kidney	Hypertensive vs. normatensive
12	GSE71994	32321	40	1050 × 1050	Blood cells	Hypertensive vs. normatensive
13	GSE87493	32321	32	1050 × 1050	Blood cells	Hypertensive vs. normatensive
14	GSE113439	32321	26	1050 × 1050	Lung tissue	Hypertensive vs. normatensive
15	GSE124114	32321	18	1050 × 1050	Endothelial Cell	Hypertensive vs. normatensive
16	GSE3356	22283	9	712 × 712	Smooth muscle	Hypertensive vs. normatensive
17	GSE11341	22283	12	712 × 712	Endothelial cells	Hypertensive vs. normatensive
18	GSE28360	32321	14	1050 × 1050	Kidney	Hypertensive vs. normatensive
19	GSE42955	32321	29	1050 × 1050	Heart	Hypertensive vs. normatensive
20	GSE67492	32321	6	1050 × 1050	Heart	Hypertensive vs. normatensive
21	GSE69601	32321	6	1050 × 1050	Blood cells	Hypertensive vs. normatensive
22	GSE70456	49495	16	732 × 732	Endothelial Cell	Hypertensive vs. normatensive

**Table 6:** Primer sequences and amplicon sizes of selected genes used in the real-time qPCR reaction.

Gene Symbol	Forward Primer	Reverse Primer	Amplicon Sizes (bp)
ADM	ATGAAGCTGGTTTCCGTCG	GACATCCGCAGTTCCCTCTT	146
EDN1	AAGGCAACAGACCGTGAAAAT	CGACCTGGTTTGTCTTAGGTG	237
ANGPTL4	GTCCACCGACCTCCCGTTA	CCTCATGGTCTAGGTGCTTGT	212
NFIL3	AGAACAAACTAATTGCACTGGGA	GCTCGTCCACAAATGAACTCAC	192
MSR1	CCAGGTCCAATAGGTCCTCC	CTGGCCTTCCGGCATATCC	94
CEBPD	CGCCATGTACGACGACGAGA	TGCTGTTGAAGAGGTCGGCG	116
USP8	GTCCAGGAGTCACTGCTAGTT	AGGAGCCAGTTTTCATAGCCT	238
GAPDH (Reference gene)	GGAGCGAGATCCCTCCAAAAT	GGCTGTTGTCAACTTCTCATGG	197

## Supplementary Materials

The following supporting information can be downloaded at: https://www.mdpi.com/article/10.3390/genes13020187/s1, Table S1: Preliminary investigation of common differentially expressed genes.
