# Supplementary material for: Quantitative Real-Time Analysis of Differentially Expressed Genes in Peripheral Blood Samples of Hypertension Patients
Source: Genes (Basel). 2022 Jan 21;13(2):187. doi: 10.3390/genes13020187 (PMC8872078; doi:10.3390/genes13020187)
Supplement: Supplementary file 1 [file genes-13-00187-s001.zip › genes-1488875-supplementary.pdf]

Quantitative Real-Time Analysis of Differential Expressed Genes in Peripheral Blood Samples of Hypertension Patients

Supplementary

Table S1. Preliminary investigation of common differentially expressed genes

Probe ID	Gene Symbol	Uniport ID	Log FC	AveExpr	t	P-Value	Adj.P.Val	B
203973_s_at	CEBPD	CEBPD_HUMAN	0.908628	7.995093	7.56545	9.56E-08	0.000304	7.334175
222802_at	EDN1	EDN1_HUMAN	1.181689	8.743519	3.416812	0.015488	1	-4.16143
203574_at	NFIL3	NFIL3_HUMAN	0.881774	6.802716	16.79369	1.36E-14	3.04E-10	18.30069
221009_s_at	ANGPTL4	ANGL4_HUMAN	2.180881	10.32553	12.18245	1.21E-11	9.01E-08	14.14263
202912_at	ADM	ADML_HUMAN	1.987131	10.64774	7.948147	4.10E-08	0.000152	8.028082
208423_s_at	MSR1	MSRE_HUMAN	-0.80507	6.478533	-5.06723	8.79E-06	0.25716	1.239517
202745_at	USP8	H0YM17_HUMAN	1.520393	8.93063	14.12079	4.21E-14	4.86E-10	22.10921
